# ‘An Emotional Earthquake’ – the Psychological Impact of the Earthquake in Syria on Mental Health Workers and the Value of Reflective Spaces: Who Cares for Carers?

**DOI:** 10.1192/bjo.2024.279

**Published:** 2024-08-01

**Authors:** Mustafa Alachkar

**Affiliations:** Mersey Care NHS Foundation Trust, Liverpool, United Kingdom

## Abstract

**Aims:**

The war-ridden northern part of Syria was struck by a powerful earthquake in February 2023 leaving thousands of people dead or injured. The consequences of the earthquake on people's mental health are harder to evaluate but are likely to be severe and long-lasting, especially as people have lived through years of war and devastation.

This poster reports on facilitating reflective practice groups, online, where Syrian mental health professionals in northern Syria explored the psychological impact of the earthquake on them as individual and as professionals.

**Methods:**

The author facilitated a series of online reflective practice group meetings. Three distinct groups of mental health workers were formed, each group consisting of 6–12 participants. Each group met twice, each session lasting an hour and a half, resulting in 6 meetings that took place between the 25^th^ of February and the 18^th^ of March 2023. In the first session the group discussed the psychological impact of the earthquake on them as individuals, and in the second the psychological impact on them as professionals.

**Results:**

Thematic analysis was conducted on the discussions in the 6 reflective group meetings, resulting in three main themes: emotional responses, cognitive responses and helpful strategies. These themes are grouped detailed in terms of the impact of the earthquake on the personal and the professional lives of the participants.

**Conclusion:**

Notwithstanding the limitations of this experience, it highlights the importance and value of group reflective spaces, as a way of helping mental health professionals process their emotional experiences in the aftermath of natural disasters.

